# MRI reveals menstrually-related muscle edema that negatively affects athletic agility in young women

**DOI:** 10.1371/journal.pone.0191022

**Published:** 2018-01-24

**Authors:** Akemi Sawai, Yuriko Tochigi, Nadzeya Kavaliova, Alexander Zaboronok, Yuki Warashina, Bryan J. Mathis, Noboru Mesaki, Hitoshi Shiraki, Koichi Watanabe

**Affiliations:** 1 Graduate School of Comprehensive Human Sciences, University of Tsukuba, Tsukuba, Ibaraki, Japan; 2 University of Tsukuba Hospital, Tsukuba, Ibaraki, Japan; 3 Faculty of Medicine, University of Tsukuba, Tsukuba, Ibaraki, Japan; 4 Faculty of Health and Sport Sciences, University of Tsukuba, Tsukuba, Ibaraki, Japan; VU medisch centrum, NETHERLANDS

## Abstract

**Context:**

About 10% of Japanese female athletes are afflicted by menstrually-related edema, mainly in the lower limbs, and, with few studies on this problem, the effect on performance remains unclear.

**Objective:**

To quantitatively evaluate fluid retention in the calf in female students over their menstrual cycle using magnetic resonance imaging (MRI) and to determine the relationship of MRI changes and athletic performance.

**Design:**

The menstrual cycle was divided into 5 phases: menstrual, follicular, ovulatory, early luteal, and late luteal with sampling done in either morning (AM) or afternoon (PM) sessions. At each phase, MRI of the calf (7:00–8:00, 14:00–16:00), body composition and hormones (7:00–8:00), and athletic performance (14:00–16:00) were evaluated.

**Participants:**

13 adult healthy Japanese female students with eumenorrhea.

**Results:**

Estradiol levels decreased significantly in the menstrual phase and the follicular phase compared to the early luteal phase (*P* = 0.001, *P* = 0.024 respectively). Menstrual phase estradiol levels were significantly lower compared to the ovulatory phase (*P* = 0.015), and the late luteal phase (*P* = 0.003). Progesterone levels decreased significantly in the menstrual phase and the follicular phase compared to the ovulatory phase (*P* = 0.012, *P* = 0.009 respectively), the early luteal phase (both *P* = 0.007), and the late luteal phase (*P* = 0.028, *P* = 0.029 respectively), and it along with a significant decrease in the ovulatory phase compared to the early luteal phase (*P* = 0.010). AM T2 signals were significantly lower in the menstrual phase compared to the ovulatory phase (*P* = 0.043) but not other phases. PM T2 signals increased significantly in the menstrual phase compared to the follicular phase (*P* = 0.003), ovulatory phase (*P* = 0.009), and the late luteal phase (*P* = 0.032), and the difference between the AM and PM values increased significantly in the menstrual phase compared to the other 4 phases (*P*<0.01). A negative correlation between fluid retention and agility was observed.

**Conclusion:**

In female students fluid retention during the menstrual phase could be a factor that influences athletic agility.

## Introduction

Currently, the number of female athletes is increasing, with the 2012 Olympic Games seeing the highest ever number of female athletes with 4675 women participating [1]. Of particular importance to supporting female athletes is investigation of the unique influence of menorrhea on physical activity and conditioning. About 80% of Japanese top-level female athletes and over 50% of regular female athletes in Japan experience menstrual discomfort [2,3] and 10% (of those 80%) are afflicted by edema [4], which are thought to be somatosensory factors that influence conditioning by increasing fear and anxiety during training. The worldwide known number of women suffering from premenstrual syndrome (PMS) differs by country and study, with 10~79% in Europe [5,6], 21~98.2% in Asia [5,7], 41% in the USA, 25.2%~97.2% in South America (Brazil) [6], and 85% in Africa (Nigeria)[5]. Previous studies have reported the prevalence of PMS as 41% of athletes and 59% of non-athletes among Iranian students [8], in Turkey, 2 studies found 55.88% and 66.11% [9], and 37.76% and 46.89% [10], respectively, and in 42.4% of female athletes in Poland [11].

In previous reports, estrogen and progesterone were suggested to have direct and indirect influence on bodily fluid deposition in tissues and sodium regulation and thereby influence menstrual cycle-related edema [12–15]. These hormones are reported to influence exercise [16,17] and athletic performance is affected by symptoms occurring before menstruation which are collectively called premenstrual syndrome (PMS) [18,19]. The indirect influence reportedly occurs through the renin-angiotensin-aldosterone system (RAAS) and changes in arginine vasopressin (AVP) secretion [14,15]. However, in the literature, the description of the exact mechanisms by which edema and its related symptoms influence female athletic performance is lacking [20].

MRI is considered a useful tool in detection of different pathological conditions in skeletal muscles that may cause an alteration in the signal intensity. Normal skeletal muscle MR signal intensity is usually slightly higher than the signal of water and much lower than that of fat on T1-weighted images and much lower than the signal of both fat and water on T2-weighted images. Muscle edema patterns, which almost always develop due to increased intracellular or extracellular water, are characterized by increased T2-signal intensity superimposed on normal appearance of the involved muscle or muscles [21].

In the current study, we aimed to quantitatively assess fluid retention associated with the menstrual cycle in healthy young women by analysis of T2 MRI signals. Such quantitative evaluation of edema associated with the menstrual cycle based on T2 signal intensity could be important to understand changes in physical condition and help to identify both the reasons for variation of water balance in the lower limbs and its effect on athletic performance.

## Materials and methods

### Study population

Initially, we analyzed data for 13 female undergraduate and graduate students (23.5±0.4 years old, 160.4±1.4 cm height) of the University of Tsukuba, Tsukuba City, Japan. The participants were recruited from May 1st to June 30th 2015. The inclusion criteria were as follows: age 20 to 25 years, menarche occurred at least 5 years before the start of the study, regular menstrual cycle, normal physical activity level (no professional athletes), physically healthy without any serious illnesses, no oral contraception or other medications, and nonsmoker. All participants received an explanation of the purpose and the flow of the study and signed an informed consent form prior to their inclusion. Powers analysis indicated that the sample size was sufficient to enable rejection of the null hypothesis. The noninvasiveness of the imaging ensured the safety of the participants. All aspects of the study were approved by the Ethics Committee of the University of Tsukuba.

### Study procedures

Starting at least 2 months prior to the MRI measurements for edema, the participants measured basal body temperature every morning after awakening and recorded it in a graphical format. Normal menstrual cycles and ovulation were confirmed by a gynecologist based on basal body temperature data analysis and luteinizing hormone surge was detected using a specific urine test (DotestLHa, Rohto Co. Ltd, Osaka, Japan). All the subjects maintained body temperature measurements until the end of the study to confirm the stability of the cycle.

The menstrual cycle was divided into 5 phases: the menstrual phase, day 1 to 4 (menses); the follicular phase, day 7 to 10; the ovulation (luteinizing hormone surge±1 day); the early luteal phase (within 7 days of the post-luteinizing hormone surge); and the late luteal phase (after the early luteal phase until menses). The measurements were carried out on one day between the first and the last days of each phase, excluding the first day of the menstrual phase (see the supplementary materials S1 Fig, S1 and S2 Tables).

In healthy individuals, no exact T2-signal values have been defined for the description of edema, which is usually related to the extension of the interstitial space, deposition of intracellular or extracellular water in various pathologies and is characterized by the increase of T2-signal intensity [21–23] In our study, the T2 signal intensity analysis was based on the method initially introduced by Bloch et al [24]. To identify the relationship between stages of the menstrual cycle and water deposition in the lower limbs, we compared the intensity of the T2 signals in the calf twice a day over the 5 phases of the menstrual cycle with the parallel measurement of the calf circumference.

**Fig 1:**
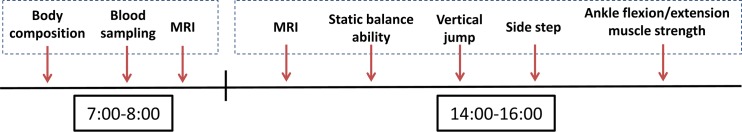
Flow of the measurements in each phase of the day.

In the morning (7:00–8:00 AM) we analyzed body composition, took blood samples for hormone analysis, measured the T2 signal intensity in the calf, and measured the calf circumference. In the afternoon (2:00–4:00 PM), we measured the T2 signal intensity in the calf, the calf circumference, and tested athletic performance (static balance, vertical jumping ability, agility, and muscle strength in ankle isometric flexion and dorsal flexion [Fig 1]). One week before initial tests and during the whole study the participants were asked to have meals on a regular basis with normal water consumption (without excessive hydration), without any alcohol, caffeine, or high-intensity physical exercises. The measurements of each of the participants’ parameters began independently with each individual’s menstrual cycle phase in random order—the first phase of the measurement of each participant being randomized to prevent habituation effect.

### Measurements

The basal body temperature was measured orally using digital thermometers (CTEB503L, CITIZEN Co, Ltd., Tokyo, Japan). A digital height meter (AD-6227, A&D Co., Ltd., Tokyo, Japan) and a body composition analyzer (Inody770, Inbody Japan, Inc., Tokyo, Japan) were used to obtain the anthropometric data on 30 indexes, including height, weight, body fat mass and volume, body water volume, lean body mass, and body mass index (BMI). Serum estradiol and serum progesterone concentrations were measured using Chemiluminescence Enzyme Immunoassay (CLEIA) and serum aldosterone concentration was measured using Radioimmunoassay (RIA).

**Fig 2:**
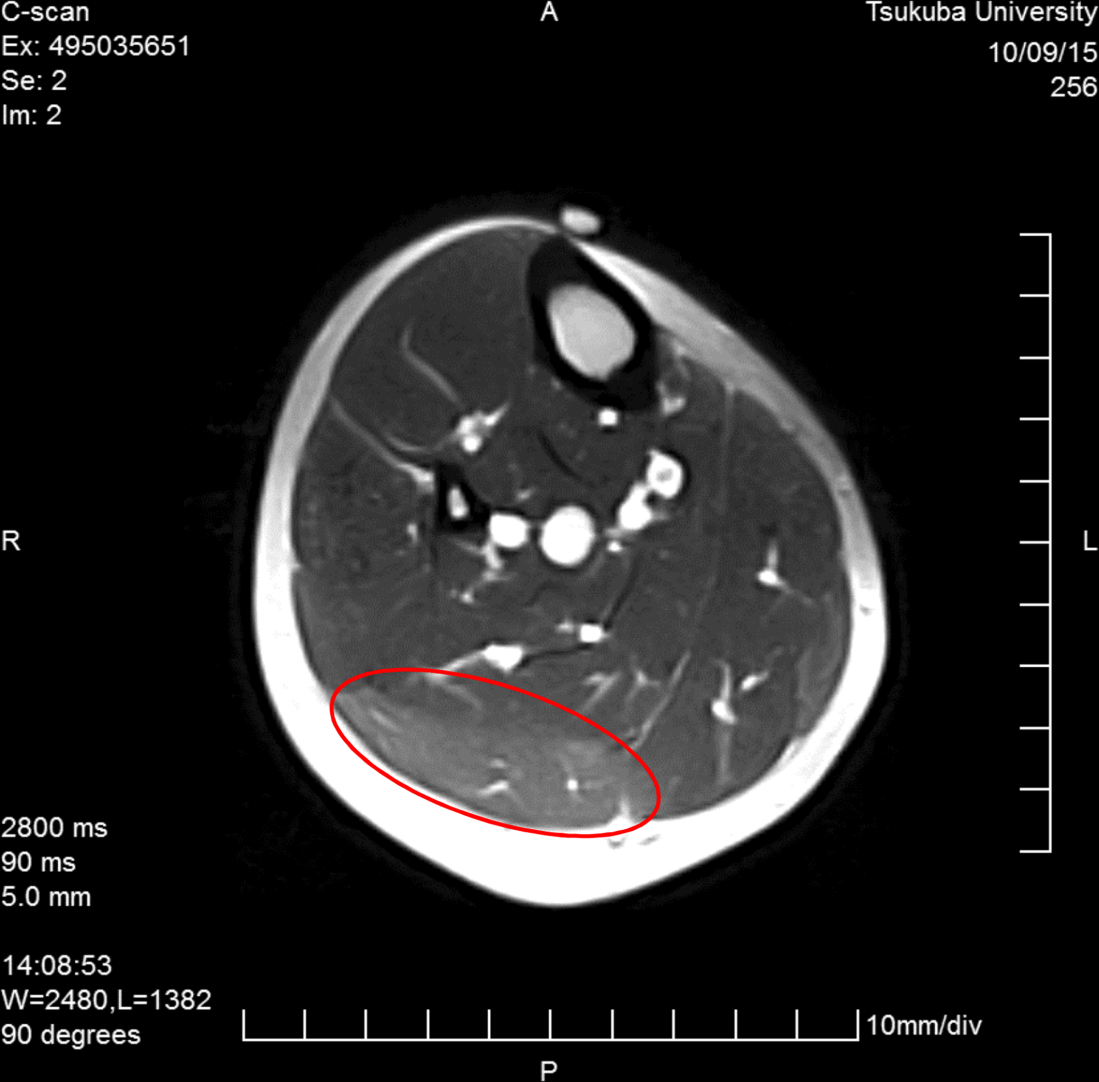
T2 signal and lateral cross-section area of the lateral gastrocnemius.

The maximum calf circumference out of 3 measurements in the dominant leg was recorded. The position of the measurement was decided on the first day and marked at a certain distance from the popliteal fossa for each individual. The T2 signal on MRI (Esaote, Inc., Napoli, Italy) was analyzed at the position of the calf’s maximum circumference. For the assessment of fluid retention, we calculated the T2 signal and the cross-sectional area of the lateral gastrocnemius (Fig 2).

The intensity of the participants’ physical activities was tracked using an activity tracker (Polar Loop, Polar Electro, Inc., Kempele, Finland) that participants wore 1 week prior to making the initial measurements through to the end of the study. Avoidance of strenuous activity was thus confirmed. We analyzed the physical activity data in 24-hour sections for every day of the 5-week study, dividing the amount of activity into 5 different degrees of exercise intensity: resting, sitting, low-, moderate-, and high-level of intensity. Additionally, we evaluated the number of steps (average step number in each phase per day) and energy expenditure (average energy expenditure in each phase per day).

Static balance ability was measured using the amplifier built-in force plate (Kistler 9286BA, Kistler Co., Ltd., Winterthur, Switzerland). Data were collected for 30 seconds, with a sampling frequency of 100 Hz and we calculated the body sway from the center-of-pressure (COP). The participants had to close their eyes, cross their arms on their chest, slightly bend their hip and knee joints and keep their balance on their dominant leg for 30 seconds. The participants practiced keeping their balance for 10 seconds 3 times before every successive measurement. If the foot of the dominant leg moved laterally or if the participants opened their eyes, or their arms detached from the chest, or the nondominant leg was used to keep their balance during the measurement, they failed the test.

**Fig 3:**
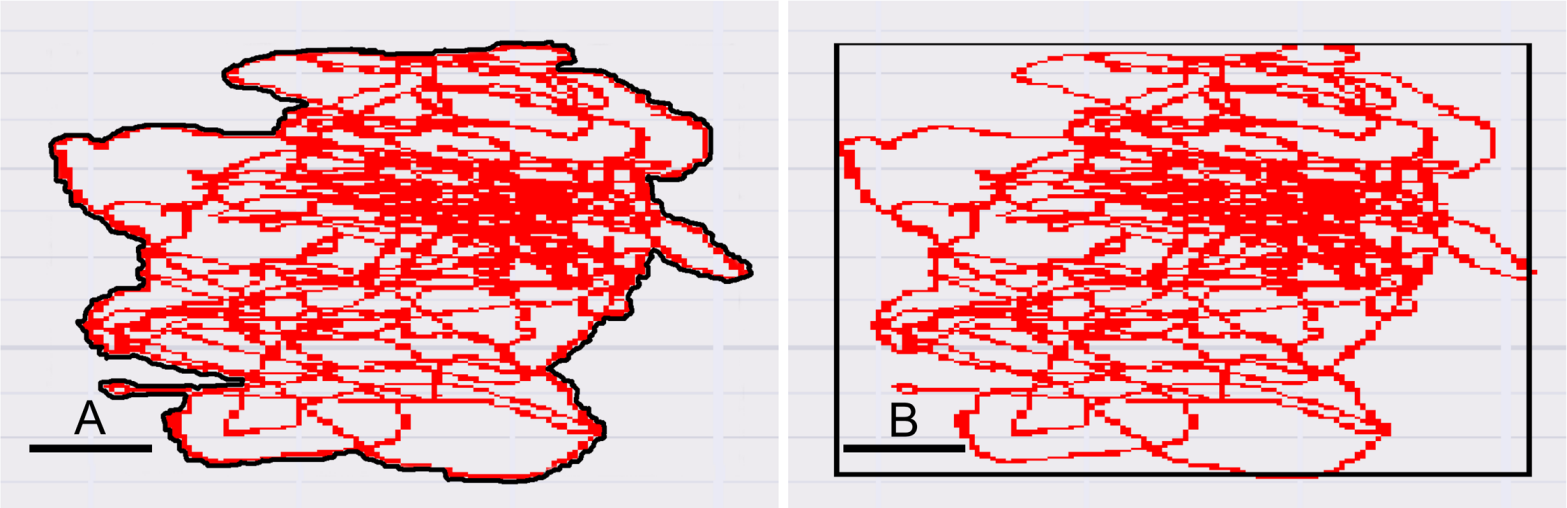
Outer peripheral area (A, cm^2^) and rectangular area of sway (B, cm^2^). Scale bar: 1 cm.

The static balance ability index was assigned as the “outer peripheral area” (cm^2^) tracing the marginal most distant balance maintaining movements, and the area of sway (rectangular) (cm^2^) limited by the farthest points of the individual balance maintaining body movements (Fig 3). Additional parameters used in the analysis were the sway area (mean circle) (cm^2^), the total locus length of the line tracing COP (cm), the total movement unit length, or the locus length per second (cm/sec), and the locus length per unit area (cm/cm^2^).

Agility was assessed using a side-step exercise, approved by the Japanese Ministry of Education Science and Technology (MEXT) as a new physical fitness test for people aged 20 to 64 [25]. In this exercise, 3 tape bands are stuck on a flat floor at 1-meter intervals. From a central starting position, the participants have 20 seconds in which to step, as many times as they can, alternately on or over the outer bands, returning to the center each time. Jumping is not allowed. If the participant steps on or over the band, it is counted as 1. If the participant fails to reach the outer band, it is counted as 0. The test is repeated 2 times for 20 seconds with a resting time of 5 minutes. We recorded the best result of the 2 tests for each individual, according to MEXT recommendations.

Vertical jumping height was measured using a digital vertical jump assessment device (Jump-MD, T.K.K 5406, Takei Scientific Instruments Co., Ltd., Niigata, Japan). The testing belt was tightly secured around the waist and the participants were told to jump as high as possible. The principle of the vertical jumping height test is similar to that of the agility test, albeit in different planes; with agility the movement is horizontal and in jumping it is vertical. The vertical jumping test was repeated twice each time, and the best result was recorded.

Ankle flexion-extension muscle strength in the dominant leg was measured by the BIODEX System 4 (Biodex Medical Systems Inc., New York, USA). Participants were in a sitting position, with their hip and the knee joints bent 90 degrees with no flexion nor extension and feet resting on a fixation plate. The thigh and the trunk of the body were immobilized with a belt and the arms were crossed at the chest. Muscle strength was evaluated by measuring the pressure on the fixation plate, at 5 seconds for flexion and 5 seconds for extension, with a 20-second rest between them. The average value from tests 2, 3, and 4, out of 5 sets, was recorded. The ankle flexion-extension test is of a different principle, where the muscle strength is evaluated and, as in previous reports where the average values of the muscle strength in different body parts was analyzed, we also followed this common principle [26,27]. The first and the last tests were excluded, as the individuals were not familiar with such tests in their everyday life and could avoid pressing the plate with 100% of their muscle strength in test 1 and could develop fatigue in test 5, unduly influencing the overall test results.

### Statistical analysis

We used SPSS version 22.0 (SPSS Inc, Chicago, IL, USA) and the repeated measures analysis of variance (RMANOVA) to evaluate differences between measured variables and correlation analysis to investigate the relationship between the T2 signal and athletic performance indexes in each of the 5 phases. All variables in the Results section were first confirmed by RMANOVA and then the post hoc tests were carried out. We performed the Bonferroni post hoc test to assess the differences among the 5 phases in each measurement. Data presented represent means±SE. *P<*0.05 was considered as statistically significant.

## Results

### Body composition and physical activity

Neither main effects nor significant changes were observed in body composition, amount of physical activity, steps, and energy expenditure over the 5 phases, suggesting that participants did not have any high intensity activities that could induce edema.

### Serum hormones concentration

The highest levels of serum estradiol and progesterone were recorded in the early luteal phase. Serum estradiol levels decreased significantly in the menstrual phase and the follicular phase compared to the early luteal phase (*P* = 0.001, P = 0.024 respectively). Additionally, the menstrual phase estradiol level was significantly lower compared to the ovulatory phase (*P* = 0.015), and the late luteal phase (*P* = 0.003). The serum progesterone level decreased significantly in the menstrual phase and the follicular phase compared to the ovulatory phase (*P* = 0.012, P = 0.009 respectively), the early luteal phase (both P = 0.007), and the late luteal phase (*P* = 0.028, P = 0.029 respectively), and it also decreased significantly in the ovulatory phase compared to the early luteal phase (*P* = 0.010). Although a main effect was observed in the serum aldosterone level and showed the highest values in the late luteal phase, there was no significant difference over the 5 phases (Table 1).

**Table 1:** Hormone levels in blood over the 5 phases.

Serum hormone concentration	Menstrual cycle															
	Menstrual phase			Follicular phase			Ovulatory phase			Early luteal phase			Late luteal phase			Main effect
**Estradiol (pg/ml)**	49.4	±	4.7^c,d,e^	79.5	±	23.6^d^	104.1	±	15.2^a^	193.8	±	23.5^a,b^	152.5	±	19.9^a^	<0.001
**Progesterone (ng/ml)**	1.1	±	0.1^c,d,e^	1.1	±	0.1^c,d,e^	2.3	±	0.3^a,b,d^	13.2	±	2.7^a,b,c^	10.6	±	2.5^a,b^	<0.001
**Aldosterone (pg/ml)**	213.2	±	31.7	234.5	±	27.9	246.8	±	39.8	295.8	±	41.3	404.7	±	79.6	0.03

The data represent means ± SEs, *P*<0.05 in a)vs. menstrual phase, b)vs. follicular phase, c)vs. ovulatory phase, d)vs. early luteal phase, e)vs. late luteal phase.

The individual estradiol and progesterone concentrations for each participant are provided in S3 Table in the supplementary materials.

### Fluid retention in the calf

There were no significant differences in the calf circumference and the cross-sectional area of the lateral gastrocnemius in the morning (AM) and afternoon (PM), and the difference between AM and PM values over the 5 phases was also insignificant (Table 2). T2 signals were significantly lower in the menstrual phase in the AM compared to the ovulatory phase (*P* = 0.043) and were not significantly different compared to other phases. In PM, T2 signal increased significantly in the menstrual phase compared to the follicular phase (*P* = 0.003), ovulatory phase (*P* = 0.009), and the late luteal phase (*P* = 0.032), and the difference between the AM and PM values increased significantly in the menstrual phase compared to the other 4 phases (*P*<0.01).

**Table 2:** Assessment of fluid retention in the calf.

Measurements	Menstrual cycle															
	Menstrual phase			Follicular phase			Ovulatory phase			Early luteal phase			Late luteal phase			Main effect
**Calf circumference (cm)**																
**AM**	34.8	±	0.5	34.6	±	0.4	34.6	±	0.5	34.6	±	0.4	34.7	±	0.4	0.703
**PM**	34.8	±	0.5	34.6	±	0.4	34.6	±	0.5	34.6	±	0.4	34.7	±	0.4	0.295
**AM-PM differ**	0.3	±	0.1	0.2	±	0.1	0.1	±	0.1	0.3	±	0.1	0.2	±	0.1	0.657
**T2 signal**																
**AM**	997.7	±	28.2^a^	1105.8	±	24.0	1107.3	±	25.4^c^	1105.1	±	30.3	1052.3	±	29.3	0.013
**PM**	1239.8	±	35.8^b,c,e^	1049.7	±	38.0^a^	1069.5	±	32.2^a^	1137.6	±	27.6	1136.7	±	34.0^a^	0.001
**AM-PM differ**	242.1	±	35.8^b,c,d,e^	-56.2	±	40.1^a^	-37.8	±	23.9^a^	32.5	±	35.2^a^	84.4	±	34.2^a^	<0.001
**Cross-sectional area (mm**^**2**^**) **																
**AM**	495.4	±	36.3	472.0	±	33.2	466.9	±	29.4	481.6	±	34.5	494.8	±	31.1	0.342
**PM**	466.3	±	32.0	480.6	±	31.4	501.3	±	37.4	478.2	±	29.8	503.4	±	27.2	0.241
**AM-PM differ**	-29.2	±	19.1	8.0	±	20.7	34.4	±	21.6	-3.4	±	16.4	8.6	±	14.1	0.193

The data represent means ± SEs, *P*<0.05 in a)vs. menstrual phase, b)vs. follicular phase, c)vs. ovulatory phase, d)vs. early luteal phase, e)vs. late luteal phase.

### Athletic performance

Athletic performance data are shown in the Table 3. The total locus length increased significantly in the menstrual phase compared to the ovulatory phase (*P* = 0.009). Other indexes of static balance ability (outer peripheral area, rectangular area, mean circle area, locus length per second, and the locus length per unit area), as well as vertical jumping ability and ankle flexion-extension muscle strength, did not change significantly over the 5 phases.

**Table 3:** Variations in the athletic performance indexes over the menstrual cycle.

Measurements	Menstrual cycle															
	Menstrual phase			Follicular phase			Ovulatory phase			Early luteal phase			Late luteal phase			Main effect
**Static balance ability**																
**Outer periphery area (cm**^**2**^**)**	14.7	±	2.7	11.7	±	1.2	11.0	±	1.1	13.6	±	1.7	13.1	±	1.2	0.055
**Rectangular (cm**^**2**^**)**	26.5	±	5.6	20.4	±	2.1	19.7	±	1.9	24.0	±	3.7	22.9	±	2.0	0.145
**Mean circle (cm**^**2**^**)**	6.2	±	1.0	4.9	±	0.5	4.8	±	0.4	5.7	±	0.7	5.3	±	0.5	0.128
**Total locus length (cm)**	185.7	±	13.3^c^	173.6	±	12.5	173.3	±	14.1^a^	180.9	±	11.6	183.3	±	8.8	0.038
**Locus length per second (cm/sec)**	6.2	±	0.4	5.8	±	0.4	5.8	±	0.4	6.0	±	0.4	6.2	±	0.3	0.078
**Locus length per unit area (cm/cm**^**2**^**)**	15.7	±	1.5	16.4	±	1.1	17.1	±	1.1	15.6	±	1.3	16.4	±	1.2	0.364
**Jump power**																
**Vertical jumping (cm)**	38.0	±	2.1	41.2	±	1.7	39.8	±	2.3	38.8	±	1.8	38.4	±	1.8	0.059
**Agility**																
**Side step (point)**	48.0	±	1.4^b,c^	51.5	±	1.5^a^	51.8	±	1.5^a, d^	50.1	±	1.6^c^	49.1	±	1.5	0.001
**Ankle flexion/extension muscle strength**																
Flexion (N*M)	86.3	±	8.5	101.1	±	7.9	99.3	±	7.9	100.7	±	8.4	100.6	±	6.1	0.055
Extension (N*M)	19.5	±	1.4	20.2	±	1.8	19.3	±	2.1	18.3	±	1.7	19.2	±	1.3	0.692

The data represent means ± SEs, *P*<0.05 in a)vs. menstrual phase, b)vs. follicular phase, c)vs. ovulatory phase, d)vs. early luteal phase.

In the side step, the results showed a significant decrease during the menstrual phase compared to the follicular phase (*P* = 0.003) and the ovulatory phase (*P* = 0.009). The highest score was recorded in the ovulatory phase. The values significantly decreased in the early-luteal phase compared to the ovulatory phase (*P* = 0.039), though there was no significant difference between the menstrual phase and the early-luteal phase. Additionally, we found a negative correlation between the T2 signal changes in the AM and PM and the side step values (correlation coefficient = -0.546, *P* = 0.025).

## Discussion

### Body composition and physical activity

Our results are in line with previous studies, which have reported that there is no significant difference in body composition over the menstrual cycle in participants who have normal physical activity level [28–30]. Thus, in our study, neither physical activity nor illness affected fluid retention in the calf and any significant differences could only be attributed to menstrual cycle influence.

### Serum hormones and fluid retention

Our results suggest that fluid retention and the difference between the AM and PM values are more prominent in the menstrual phase. Furthermore, the variations between the AM and PM regarding the fluid component increase gradually from the late luteal phase to the menstrual phase and then gradually decrease after the menstrual phase and continue to the follicular phase, showing less fluid variations in the ovulatory phase.

In previous reports, edema was assessed by measuring calf circumference or by subjective evaluation [31,32]. In these reports, fluid retention, assessed using a subjective scale, peaked on the first day of menstruation [33,34], which agrees with our results. Our findings also support the suggestion that the edema could be the result of a delayed response to the previous higher hormone levels [34]. The reason for this still remains unclear, however, the increase in the serum hormone levels and the appearance of the edema with the 3–4 days delay is obvious.

Although participants of the present study did not complain of any typical PMS symptoms, the results show that fluid retention increased significantly during the menstrual phase. Thus, we found that edema occurred regardless of subjective symptoms. Furthermore, T2 signal changes during the menstrual cycle showed fluid retention when the levels of estrogen, progesterone, and aldosterone were the lowest, which contradicts the main theory of premenstrual edema.

A decrease in physical activity level may be a reason for the occurrence of edema. Although, during the present study, there was no significant difference in the physical activity level over the menstrual cycle, there is a possibility that participants may unconsciously have a decrease in physical activity level due to menstruation. Saito et al reported that over 60% of the female participants in their study complained of lethargy during menstruation and over 40% answered that they became depressed [35]. In our study, we collected data on specific symptoms (PMS) over the menstrual cycle without assessing the mood component. Nevertheless, it is possible that related mechanisms somehow influence both mood and fluid retention during the menstrual phase.

### Athletic performance

Hayashi et al reported that static balance ability decreased significantly in the menstrual, early-luteal, and late-luteal phases compared to the follicular phase and the locus length per second decreased significantly in the late-luteal phase [36], suggesting the secretion of estradiol and progesterone as a possible cause. These hormones are also reported to have a central nervous system effect, potentially affecting posture control indirectly [37–39]. In the present study, only one additional parameter in the static balance ability decreased significantly in the menstrual phase without significant changes in the late-luteal phase. We speculate that the menstrual bleeding itself might also be the reason for the impaired static balance ability. Moreover, we found no correlation between fluid retention and static balance ability.

Giacomoni et al showed that jumping power decreased significantly in the menstrual phase in athletes using the multi-drop jump and the squat jump [40]. We did not use these tests, as they carry some risk of injury for non-athletes. However, even with a digital vertical jump assessment device it was quite difficult for participants to jump accurately and vertical jumping could also depend on arm movement much more than lower extremity mechanical power [41], which could account for our results.

Lebrun et al reported that the best agility performance was generally recorded in the first postmenstrual days, with the worst performance during the premenstrual interval and the first few days of the menstrual flow, which agrees with our results [18]. Hashimoto et al assessed agility in female handball players and reported that the agility was lowest in the menstrual phase and best in the follicular phase [42]. However, the 25m shuttle run they used needed technical skills and no measurements were done in the ovulatory, early-luteal, and late-luteal phases. In Japan, the side step exercise is generally used in physical education classes to assess agility and was familiar to the participants of our study and they had a practice session before the measurement. Therefore, we suggest that the results of our agility test were influenced only by the menstrual cycle itself.

Some previous reports have shown a relationship between muscle strength in the legs and the menstrual cycle in athletes, in which muscle strength increased mostly during the follicular phase [26,27, 43,44]. Our results are in line with the study by Jonge et al, who found no significant difference in knee flexion-extension muscle strength (isokinetic) over the menstrual cycle in women with normal physical activity level [45]. Thus, we suggest that the difference in the physical activity level in participants (athletes versus non-athletes) may lead to a difference in muscle strength over the menstrual cycle.

Our objective study was the first to investigate that body fluid accumulation varies over the 5 menstrual phases in spite of the lack of subjective feelings of swelling. Although there was no significant correlation between the results in the MRI T2 signal and the ovarian hormone levels, there was a significant difference over the 5 phases in diurnal body fluid variation which was evaluated by the MRI T2 signal. We speculate that fluid accumulation occurrences are directly or indirectly affected by the ovarian hormones and may negatively influence both athletic performance ability (especially agility which was assessed by the side-step in our study) and musculoskeletal treatment of injuries in the clinical setting. Although our study was, for practical purposes, restricted to the gastrocnemius, evaluation of hormonal effect on fluid retention within muscle tissue is highly important clinical knowledge because there could be implications in treating muscular injuries that may be overlooked by subjective examination. That is to say, even if the patient is not aware of edema, the physician can ask about menstrual status and will now have objective measuring tools to check for any edema that could affect subsequent clinical treatment options [46].

Considering possible limitations of this study, a larger number of subjects would be beneficial for more precise evaluation of the studied parameters, though taking into account the novelty of the approach we proposed, our early stage work may lead to further development of the method and an increase in the number of future studies. Longer periods of hormone level evaluation and test exercises would provide data for multiple menstrual cycles for more sustained analysis. The prevalence of menstrually-related muscle edema is familiar in female athletes though the description of such edema and data on its influence to the athletic performance is lacking. In female athletes, it may be hard to discriminate whether the variation of water retention occurs due to either sex hormone fluctuations or as a response to exercises and, at this stage, healthy individuals were included in our study. Recruitment of female athletes along with women with normal physical activity levels will be favorable for this project, which at the moment is a first step in accumulating base-line data for further investigation of the phenomenon in athletes.

## Conclusion

Fluid retention in the legs (T2 signal intensity) increased significantly during the menstrual phase in the afternoon, and occurred regardless of subjective symptoms. Side-step ability showed a significant decrease during the menstrual phase and had a negative correlation with fluid retention (T2 signal intensity) in the legs. These might be factors that negatively influence the physical performance of women and should be considered in further sports-related studies.

## Supporting information

S1 Fig Measurement days within early luteal (EL) and late luteal (LL) phases (Example Subject A). The measurements were carried out on one day between the first and the last days of each phase, excluding the first day of the menstrual phase.
(TIF)

S1 Table The day of the test counted from the first day of the menstruation in each participant (n = 13). (XLSX)

S2 Table The length of the cycle/luteal phase and measurement days in early luteal (EL) and late luteal (LL) phases in all subjects. (XLSX)

S3 Table The individual estradiol and progesterone concentrations for each participant in each phase of the menstrual cycle. (XLSX)
