# Supplementary figures and images for: MRI reveals menstrually-related muscle edema that negatively affects athletic agility in young women
Source: PLoS One. 2018 Jan 24;13(1):e0191022. doi: 10.1371/journal.pone.0191022 (PMC5783373; doi:10.1371/journal.pone.0191022)

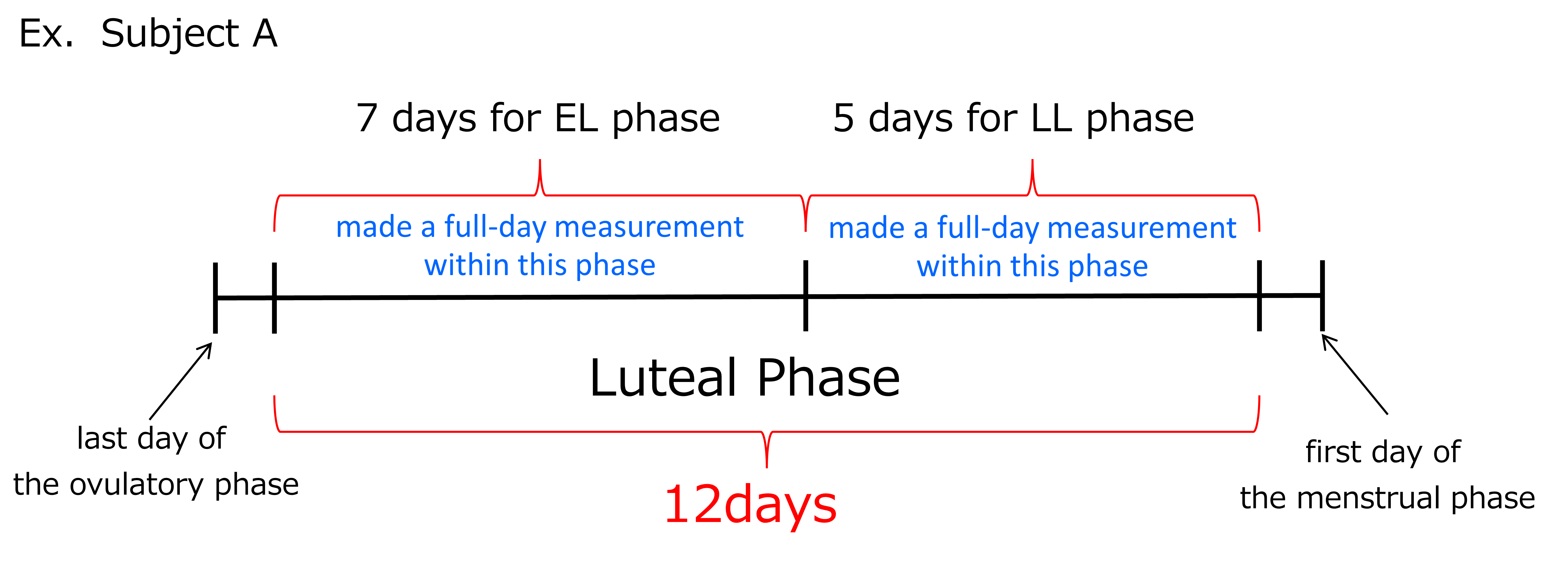

Supplement: S1 Fig — The measurements were carried out on one day between the first and the last days of each phase, excluding the first day of the menstrual phase. (TIF) [file pone.0191022.s001.tif]
